# Secondary Polycythemia and the Risk of Venous Thromboembolism

**DOI:** 10.14740/jocmr1916w

**Published:** 2014-07-28

**Authors:** Vijaya Raj Bhatt

**Affiliations:** Division of Hematology-Oncology, Department of Internal Medicine, University of Nebraska Medical Center, 987680 Nebraska Medical Center, Omaha, NE 68198-7680, USA. Email: vijaya.bhatt@unmc.edu

## To the Editor

With an increasing incidence of obesity, sleep apnea and other chronic pulmonary diseases, the prevalence of secondary polycythemia is expected to rise in adults. Phlebotomy is commonly utilized in the management of secondary polycythemia. Although supported by a number of studies demonstrating a transient improvement in cardiovascular and cerebrovascular physiology, the benefit of phlebotomy is unclear with the possible exception in patients with hyperviscosity symptoms. The popularity of phlebotomy also reflects the overgeneralization of its survival advantage in polycythemia vera. Polycythemia vera, frequently accompanied by thrombocytosis, leukocytosis and enhanced adhesion of blood cells and endothelial cells, have an enhanced risk of thrombosis. Conversely, the risk of thrombosis associated with secondary polycythemia is uncertain.

Using search terms, “secondary erythrocytosis” or “secondary polycythemia” and “thrombosis” or “embolism”, I reviewed studies evaluating the risk of arterial or venous thrombosis in adult patients with secondary polycythemia. Following inclusion criteria were used: English-language studies, published and PubMed-indexed between 1990 and November 2013, adult patients, and polycythemia secondary to cardiac or pulmonary disease, smoking or idiopathic causes. Case reports were excluded. Five observational studies have determined the risk of thrombotic complications (Table 1). Three studies failed to demonstrate an elevated risk of arterial or venous thrombosis with secondary polycythemia [1-3]. A prospective study showed an increased risk of pulmonary embolism with polycythemia; however, the study included patients with respiratory distress and elevated D-dimer, did not match groups with and without polycythemia, and did not assess smoking history [4]. A retrospective case-control study from 1993 revealed a higher incidence of arterial and venous thrombosis in polycythemia vera (60%) compared to smoker’s polycythemia (41%). Such high incidence of thrombotic complications in secondary polycythemia group was the result of arterial events (92%), which is explained by the history of smoking and the possibility of undiagnosed underlying myeloproliferative disorder [5].

**Table 1:** Secondary Polycythemia and the Risk of Venous Thromboembolism in Adults

Author, year	Study groups (N)	Age in years	Etiology of PCT	Definition of PCT	Average HCT	Outcomes	Strength	Limitation
Ristic et al, 2013 [4]	PCT (100) vs. non-PCT (262)	66 vs. 63	COPD/respiratory failure	HCT > 50%	58% vs. 40%	PE 39% vs. 11% (P < 0.001); DVT 5% vs. 4% (P = 0.87)	Only prospective study	Inclusion criteria,* unmatched groups, smoking habits not assessed
Nadeem et al, 2013 [1]	PCT (86) vs. non-PCT (86)	68 vs. 68	COPD	HCT ≥ 50% on two occasions	53% vs. 43%	VTE 19% vs. 14% (P = 0.42) †	Well-matched groups	No data on the use of phlebotomy
Perloff et al, 1993 [2]	Comp (101) vs. Uncomp (11) PCT	36 (19 - 74)	Cyanotic CHD	HCT > 45%	57% vs. 69%	Stroke 0% ‡	High-risk patients with hyperviscosity symptoms; long follow-up	Phlebotomy at 3 - 6 months interval for symptom relief (20% vs. 80%)
Schwarcz et al, 1993 [5]	SP (27) vs. PV (43)	55 vs. 59	Smoking vs. PV	Increased RBC volume	59% vs. 59%	Arterial or venous thrombosis 41% vs. 60% (P < 0.05) §	PV is more hypercoagulable than SP	Diagnostic criteria, smoking as a confounder
Lubarsky et al, 1991 [3]	PCT (100) vs. non-PCT (100)	57 vs. 60	COPD	Hgb > 16 g/dL	Hgb 17 vs. 13 g/dL	Arterial or venous thrombosis 0% vs. 3%; (P = NS)	No thrombotic complications even during surgery	Follow-up limited to 30 days postoperatively; significant postoperative blood loss in 25% patients with PCT

CHD: congenital heart disease; Comp: compensated; COPD: chronic obstructive pulmonary disease; DVT: deep venous thrombosis; HCT: hematocrit; Hgb: hemoglobin; N: number of patients; NS: not significant; PCT: polycythemia; PE: pulmonary embolism; PV: polycythemia vera; RBC: red blood cell; SP: secondary polycythemia; Uncomp: uncompensated; VTE: venous thromboembolism. All studies, except by Ristic et al [4], were retrospective. *The study included patients who presented with respiratory distress, and were found to have elevated D-dimer. †Obesity was identified as a possible risk factor for venous thromboembolism in secondary polycythemia. ‡A 28-year-old female with iron-deficient polycythemia had four episodes of amaurosis fugax during few months when hematocrit level ranged between 63% and 73%. §In smoking-related secondary polycythemia, there was only one venous thromboembolism and 11 arterial events (myocardial infarction, stroke or transient ischemic attacks). Risk of venous thromboembolism was 4% vs. 21% in secondary polycythemia compared to polycythemia vera. The incidence of recurrent events was higher in polycythemia vera compared to secondary polycythemia (28% vs. 7%). Within secondary polycythemia, patients who had an event had lower hematocrit and platelet count than those who did not have an event.

In conclusion, there is no definite evidence that secondary polycythemia *per se* increases the risk of thromboembolism. Taken together with the transient nature of any beneficial effects, concerns for potential risks such as hypotension and iron deficiency, phlebotomy should not be routinely utilized in the management of secondary polycythemia until it is supported by well-designed studies in the future. Future studies should also assess the possibility of increased thrombotic complications in certain subsets of secondary polycythemia such as those with additional risk factors, e.g., obesity [1] or varicose vein [4], and the possibility of an elevated risk of unprovoked venous thromboembolism [1, 4].

## References

[1] Nadeem O, Gui J, Ornstein DL (2013). Prevalence of venous thromboembolism in patients with secondary polycythemia. Clin Appl Thromb Hemost.

[2] Perloff JK, Marelli AJ, Miner PD (1993). Risk of stroke in adults with cyanotic congenital heart disease. Circulation.

[3] Lubarsky DA, Gallagher CJ, Berend JL (1991). Secondary polycythemia does not increase the risk of perioperative hemorrhagic or thrombotic complications. J Clin Anesth.

[4] Ristic L, Rancic M, Radovic M, Ciric Z, Kutlesic KD (2013). Pulmonary embolism in chronic hypoxemic patients with and without secondary polycythemia-analysis of risk factors in prospective clinical study. Med Glas.

[5] Schwarcz TH, Hogan LA, Endean ED, Roitman IT, Kazmers A, Hyde GL (1993). Thromboembolic complications of polycythemia: polycythemia vera versus smokers' polycythemia. J Vasc Surg.

